# Pregnancy following kidney transplantation - impact on mother and graft function and focus on childrens’ longitudinal development

**DOI:** 10.1186/s12884-019-2496-z

**Published:** 2019-10-23

**Authors:** Friederike Bachmann, Klemens Budde, Marie Gerland, Cornelia Wiechers, Nils Heyne, Silvio Nadalin, Sara Brucker, Cornelia Bachmann

**Affiliations:** 1 Department of Nephrology and Medical Intensive Care medicine, Charité University medicine Berlin, Chariteplatz 1, 10117 Berlin, Germany; 2 Department of Obstetrics and Gynecology, University Women’s Clinic, University of Tuebingen, Calwerstrasse, 7, 72076 Tuebingen, Germany; 3 Department of Neonatology, University Children’s Hospital Tuebingen, Calwerstrasse 7, 72076 Tuebingen, Germany; 4 Department of Endocrinology and Diabetology, Angiology and Nephrology and Clinical Chemistry, University of Tuebingen, Otfried-Müllerstr. 10, 72076 Tuebingen, Germany; 5 Department of General-, Visceral- and Transplant Surgery, University Hospital of Tuebingen, Tuebingen, Germany

**Keywords:** Pregnancy, Kidney transplantation, Children development, Preterm birth

## Abstract

**Background:**

Pregnancy after kidney transplantation has been considered as high risk for maternal and fetal complications. After careful patient selection successful pregnancies are described. Little is known about fetal outcomes and data is particularly scarce on childrens´ early development up to two years when born to kidney/−pancreas transplant recipients.

Here, we analyzed maternal and fetal risk and evaluated graft function during pregnancy in transplanted women. We aimed to identify factors affecting the outcomes of mothers and their grafts during pregnancy and of children up to 2 years after delivery/ birth.

**Methods:**

All consecutive pregnancies in kidney/ kidney-pancreas recipients with live-born children from 2002 to 2016 were evaluated in two transplant centers (Charité Berlin/ University Tuebingen). All data was gathered from medical records. Impact of pregnancy on obstetrical risks, graft function and fetal development was evaluated. Additionally, for the first time development of children, including physical examination and assessment of neurological function were evaluated at 12 and 24 months.

**Results:**

Thirty-two pregnancies in 28 patients with a median duration of 34 gestational weeks (range, 24–38) were analyzed. 13 patients (46.4%) developed deterioration of kidney graft function > 10 ml/min during pregnancy. In majority, caesarean section was performed (75%). Twenty-five (78.1%) children were born prematurely, thereof (16%) < 28 weeks. Almost 70% had low birth weights (LBW) (< 2.500 g); median birth weight was 2.030 g. General health and physical constitution of children were unremarkable with normal development in 94% at 12 and 24 months of corrected age, respectively.

**Conclusion:**

Despite the high rate of preterm birth and LBW, development up to two years was age-appropriate in this cohort. Due to low absolute numbers, increasing efforts in centralized counseling, diagnostics and committed specialist support are required. Decisive treatment of these high-risk patients in specialized units leading to better performance of these patients (mother/ fetus) is deemed superior. In order to confirm this, prospective studies on neonatal and pediatric outcomes with a standard-of-care comparator arm will be conducted.

## Introduction

There is an increasing number of positive reports on (successful) pregnancies in kidney transplant recipients [1–5]. Nevertheless, pregnancies under these specific conditions are considered as high risk especially concerning unavoidable treatment of immunosuppressive drugs, underlying kidney disease and other comorbidities. Indeed, complications are relatively common and this should be reflected in patient counselling and clinical decision making [6–8]. Among women after renal transplantation the risk of pregnancy-related complications, including preeclampsia/ gestational diabetes, caesarean section, prematurity, fetal growth restriction and low birth weight compared to general population are significantly increased in this subgroup [7, 9].

It was recently shown in a cohort of 56 female transplant recipients that adequate graft function (creatinine < 110 μmol/l) was positively correlated with pregnancy-related complications such as preeclampsia and preterm delivery [8].

Prematurity is associated with several acute and chronic complications including respiratory distress syndrome, intracranial hemorrhage, apnoea, retinopathy of prematurity, seizures, necrotizing enterocolitis and temperature instability [10]. These infants are at high risk of long-term neurodevelopmental morbidities such as cerebral palsy, mental disorders and impaired learning [11, 12].

Interestingly, data on children’s early development in pregnancies after kidney transplantation are scarce even though many reports on successful pregnancies in kidney transplant recipients are available [3–8]. Even more, mid- or long-term development and health of children born to kidney transplanted mothers is a subject less studied [13, 14]. The most recent analysis of children born to transplanted mothers evaluated cognitive development. Data suggest that their cognitive development is no different from that of controls [15]. An earlier National Transplant Pregnancy Registry analysis of children exposed in utero to cyclosporine, revealed no increased risk of birth defects, significant problems with renal function, attention-deficit hyperactivity disorder or neurocognitive or immune development [16]. These findings are in accordance with results from previous studies that show a high rate of adequately developed children born to kidney transplanted mothers [13, 14].

The aim of this multi-center study was to evaluate pregnancies following kidney transplantation and their impact on mother, graft and fetus/ early childhood with observation up to 2 years after delivery.

## Patients and methods

### Study design and population

From January 1st 2002 through 31th December 2016, we evaluated retrospectively all 28 consecutive patients with pregnancy and delivery after kidney or simultaneous kidney-pancreas transplantation at University Hospital Tuebingen and Charité University Berlin. Only pregnancies resulting in birth > 23 weeks of gestation were included.

Mycophenolate (MPA) was routinely used for rejection prophylaxis in both centers since 1996. MPA was routinely discontinued at least 4 weeks prior to planned pregnancy and replaced by steroids or azathioprine, respectively. The decision to either steroids or azathioprine was left at discretion of the treating physician.

After confirmation of pregnancy clinical parameters were recorded at regular intervals by department of gynecology and nephrology. The following characteristics were analyzed: age at transplantation/ delivery, kidney function pre- pregnancy/ at one and six months/ at one year and up to two years after delivery were gathered. Pre-pregnancy creatinine was defined as the latest result within three months before pregnancy. Glomerular filtration rate (GFR) was calculated according to the 2009 Chronic Kidney Disease Epidemiology Collaboration (CKD-EPI) equation [17]. Time interval from transplantation to pregnancy was set to the estimated date of conception and in case of several pregnancies it was calculated separately for each pregnancy. Graft loss after pregnancy was defined as returning to dialysis or transplantation. We also recorded the following parameters: cause of end-stage renal disease (ESRD), donor type (living/ deceased donor), type of maintenance immunosuppressive therapy, occurrence of pre-pregnancy hypertension (defined as preexisting intake of antihypertensive treatment or hypertensive blood pressure profiles) and other complications like new-onset hypertension, preeclampsia (diagnosed according to revised recommendations of American Congress of Obstetricians and Gynecologists) [18], gestational diabetes and incidence of caesarean section. Indications for caesarean deliveries were subdivided into three categories: maternal, fetal and combined fetal/maternal. Maternal indications consisted of severe hypertensive disorder in pregnancy, pre-eclampsia, deterioration of kidney graft function including urine retention and increase in proteinuria or amniotic fluids disorders, prolonged labor and previous caesarean delivery. Fetal indications included intrauterine growth restriction, fetal distress like cardiotocogram abnormalities and fetal malposition; complications related to both, mother and fetus, such as prolonged labor and chorioamnionitis. Gestational age was calculated in weeks starting from the first day of the patient’s last menstrual period. In presence of an early ultrasound gestational age as calculated from that.

To determine the association between outcomes and the interval between kidney transplant and pregnancy, we defined the following groups: <two years versus two to five years versus more than 5 years.

Additionally, physical and psychomotor development of the children in our cohort up to 2 years were examined by a pediatrician. Specifically, neonatal outcome was observed, including birth weight, gestational age, intrauterine growth restriction, APGAR -score (Appearance, Pulse, Grimace, Activity, Respiration), malformation. In order to achieve age-related variables, corrected age based on expected date of birth in preterm deliveries was used. Data were collected from the patient’s medical records. Weight was measured with an electronic scale accurate to the nearest 1 g. Length was measured with an infant-length board to the nearest 1 mm when the weight was > 1.000 g or with a nonstretch measuring tape for very preterm infants in a closed incubator. Head circumference was measured in the largest frontooccipital plane to the nearest 1 mm with a nonstretch measuring tape. Weight, length and head circumference were converted to age-specific and gender-specific Z-scores according to the WHO growth chart using calculator (Anthro software).

Additionally, all mothers after live birth were asked to complete a questionnaire on their childrens’ physical developmental status. The questionnaire contained physical examination, anthropometric measures, medical and paramedical history. These findings were derived from medical examination by the pediatrician. It was complemented by regular pediatric exams of functions and abilities at 12 and 24 months (± 2 months) the results of which were reported within the questionnaires.

Neonates were categorized according to World Health Organization defined as extreme premature (< 28 weeks of gestation), very preterm (28–32 weeks) and moderate-to-late preterm (32–37 weeks) [19]. They were also categorized according to their birth weight as defined by World Health Organization as low birth weight (LBW; < 2.500 g) regardless of gestational age at time of birth.

Based on gestational age, percentile values for birth weight, birth length and head circumference for girls and boys were calculated according to Voigt et al. [20]. Neonatal death was defined as dying within 28 days after delivery. Based on gestational age-adjusted birth weight, birth length or head circumference, percentiles are calculated for infants classed as small for gestational age (SGA), appropriate for gestational age (AGA, 10th – 90th percentile) or large for gestational age (LGA). A birth weight < 10th percentile is described as SGA; a birth weight > 90th percentile is described as LGA. Fetal growth restriction (FGR) is defined as growth <10th percentile and pathologic doppler- ultrasound of umbilical artery or uterine artery.

### Statistics

Data are reported as mean (standard deviation) or median [interquartile range (IQR)] for skewed data. All statistical analyses were performed using SPSS version 25 (SPSS Inc., Chicago, IL, USA). Continuous variables were compared using Student t-test. Graft and patient survival were analyzed by Kaplan-Meier analysis. Values of *p* < 0.05 were considered as statistically significant.

## Results

Over the 14-year period 28 women had 32 pregnancies (> 23 gestational weeks) resulting in 32 live births, including five women (17.8%) who had two live births*.*

### Maternal characteristics

**Fig. 1:**
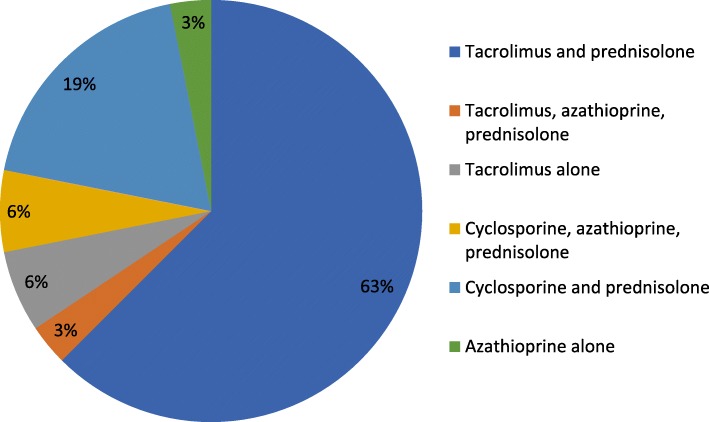
Immunosuppressive regimens during pregnancy

26 women had received only a kidney transplant; two women a simultaneous kidney-pancreas transplantation. Table 1 summarizes the baseline maternal characteristics. Twenty-three patients were recipients of a first kidney transplant, two of a second transplant, and one patient got a third transplant. Almost 50% of patients (12/28, 46.1%) were recipients of living related donor kidney transplant (Table 1). The most common primary maternal diagnosis was chronic glomerulonephritis (11/28, 39.2%). Age at transplantation was 26.3 years in median (IQR 22.3, 29.9) and 32.8 years (IQR 29.3, 35.3) at delivery. Pregnancies occurred at a median of 5.9 years (IQR 3.3, 8.6**)** after transplantation, 65.6% of pregnancies occurred > 5 years after transplantation. Hypertension was present in 67.8% of women before pregnancy. Immunosuppressive regimens for the 32 pregnancies are shown in Fig. 1**.** Maintenance immunosuppression consisted of calcineurin-inhibitor (tacrolimus (*n* = 20) or Cyclosporine A (*n* = 6)) and steroids as dual therapy in majority of pregnancies (26/32, 81.2%). Three women received triple immunosuppression in combination with azathioprine (Fig. 1). One woman received azathioprine monotherapy and two were on monotherapy with tacrolimus (Fig. 1). In the majority of the women (89.3%) MPA was stopped prior to planned pregnancy and in three women (10.7%) after confirming pregnancy.

**Table 1:** Maternal characteristics of kidney transplant recipients with pregnancy

Patients	*n* = 28(%)*
Age at transplantation (years)	26.4 ± 5.6
Age at delivery (years)	32.0 ± 4.3
Number of kidney transplantations	
1st kidney transplant	23(82.2)
2nd kidney transplant	2(7.1)
3rd kidney transplant	1(3.6)
Kidney-pancreas transplant	2(7.1)
Living kidney donor	12(46.1)
Deceased donor	16(53.9)
Cause of end-stage renal disease	
Glomerulonephritis	11(39.3)
Diabetes type I	2(7.1)
Hemolytic uremic syndrome	2(7.1)
Obstructive nephropathy	2(7.1)
Interstitial nephritis	2(7.1)
Nephronophthisis	2(7.1)
Vesicourethral reflux	2(7.1)
Unknown	5(17.9)
Interval from transplantation to conception**	
< 2 years	2(6.1%)
2–5 years	9(27.2%)
≥ 5 years	21(65.6%)
Pre-pregnancy hypertension	19(67.8)
Number of antihypertensive agents pre-pregnancy, median	2

*26 renal transplanted women and two simultaneous kidney-pancreas transplanted women

**five patients had two pregnancies. Each pregnancy was calculated separately

Most of the pregnancies occurred more than 5 years after transplantation (Tables 1,2).

**Table 2:** Pregnancy outcomes stratified by interval between transplantation and conception

Time interval (years) Transplantation-conception	> 2–5 years (*n* = 9) (%)	> 5 years (*n* = 21) (%)	*P* value
Median maternal age at delivery (years, IQR)	32.8(29.5, 33.5)	32.9(29.0, 36.0)	0.533
Preeclampsia	0	5(23.8%)	0.021
Caesarean section	5(55.5%)	18(85.7%)	0.075
Preterm birth	5(55.5%)	17(80.9%)	0.078
Gestational age (weeks), Median (IQR)	36.29 (34.7, 37.5)	34.3 (29.6, 36.1)	0.030
Birth weight (g) Median (IQR)	2.280(1.940, 2715)	1.885(985, 2290)	0.058
Low birth weight	5(55.5%)	17(80.9%)	0.078

Evaluation of 32 pregnancies (thereof 5 patients with 2 pregnancies) subdivided in 2 groups according to transplant interval. Values are presented as median (interquartile range, IQR)

The comparison of interval between > 2-5 years and > 5 years from transplantation to pregnancy leads to following results (Table 2).

The gestational age median was 36.29 weeks in patients at two to five years interval after transplantation vs 34.29 weeks in patients > 5 years after transplantation.

The highest rate of preterm birth occurred in the interval between transplantation and pregnancy > 5 years associated with low birth weight in 80.9% (Table 2) without significant difference. A significantly higher rate of preeclampsia was seen in the group > 5 years compared to the group > 2-5 years (*n* = 5; *p* = 0.021; Table 2). None of the patients in the group > 2-5 years had preeclampsia.

### Duration of gestations and delivery mode

Duration of all 32 enrolled pregnancies was 34.7 gestational weeks in median (IQR 31.9, 36.3, Table 3**)**. 78.2% of children (*n* = 25) were born prematurely, thereof four (12.5%) were born < 28 weeks because of maternal infection or early contractions (Table 4). In 24/32 pregnancies (75%) a caesarean section was performed, thereof two patients received a caesarean section with labor; 8 patients (25%) had a vaginal delivery (Table 3). Six patients had already undergone caesarean section before transplantation. Most often (66.7%), primary caesarean section was performed due to maternal conditions, in 5 (20.8%) patients due to fetal conditions and in 3 (12.5%) patients due to combined fetal/maternal conditions (Table 3). Of those patients with primary caesarean due to maternal conditions 5 patients (20.8%) had preeclampsia. One of those developed aortic dissection due to worsening of hypertension and an emergent caesarean section was performed in the 30th gestational weeks. For those in the fetal/maternal indication category one had placental abruption and two had placental insufficiencies; FGR and fetal distress during labor were fetal indications for caesarean in 5 cases (Table 3). No graft injuries occurred during delivery.

**Table 3:** Pregnancy outcome until 2 years after delivery

Patients	n = 28
Number of pregnancies	32
Median gestational age (weeks), IQR	34.7(31.9, 36.3)
Delivery	
Vaginal birth	8(25%)
Caesarean section without labor*	22(68.8%)
Caesarean section with labor	2(6.2%)
Preeclampsia	5(15.6%)
Gestational diabetes	2(6.2%)
Indication for caesarean section	
Maternal	16(66.7%)
Fetal	5(20.8%)
Combined (maternal and fetal)	3(12.5%)
Renal outcome	
Pre-pregnancy GFR _(CKD-EPI)_ (ml/min/1,73m^2^), median (IQR)	57(52, 78)
GFR _(CKD-EPI)_ at delivery	49(37, 59)^a^
GFR _(CKD-EPI)_ 12 months after delivery	50(39, 69)^b^
GFR _(CKD-EPI)_ 24 months after delivery	47(41, 80)
Graft failure within 24 months after delivery	2(6.2%)
Proteinuria	
Proteinuria at delivery, mg/g creatinine, median (range)	335(100–4000)

All 32 pregnancies resulting in life births are evaluated. The indication for caesarean section is evaluated in 24 pregnancies and is subdivided in three groups (maternal/ fetal/ combined maternal and fetal). GFR, glomerular filtration rate. IQR, interquartile range

*two emergency caesarean section due to aortic dissection due to severe hypertensive disorder and major placental abruption

eGFR _(CKD-EPI)_, estimated glomerular filtration rate. ^a^P 0.005 and ^b^P 0.001 versus GFR _**(CKD-EPI)**_ at pre-pregnancy

**Table 4:** Characteristics of newborn

Live births	*n* = 32(%)	P value
Median gestational age (weeks), **(**IQR)	34.7(31.9, 36.3)	
Live births (gestational age, weeks)		
Extremely premature birth (< 28 weeks)	4(12.5)	
Very premature birth (28–32 weeks)	4(12.5)	
Moderate- to late premature birth (32–37 weeks)	17(53.1)	
Term > 37	7(21.8)	
Median birth weight (grams, IQR)	2030 (1380, 2375)	
SGA	13(40.6)	
AGA	19(59.6)	
Birth weight (grams)		
3000–3500 g	2(6.1)	
2500–3000 g	5(15.6)	
< 2500 g, low birth weight*	25(75.7)	
Median birth weight (grams, IQR)		0.002
Preterm	1810(1105, 2240)	
Term	2550(2225, 2915)	
Median Birth length (cm, IQR)		0.006
Preterm	43.0(37.0, 46.0)	
Term	48.0(46.0, 50.0)	
Head circumference (cm, IQR)		0.011
Preterm	31.0(27, 32.5)	
Term	33.0(32.0, 34.0)	
pH at delivery (livebirths)		
< 7.0	0	
> 7.0- ≤ 7.1	1(3.1)	
> 7.1- ≤ 7.2	8(25)	
> 7.2	23(71.8)	
APGAR		
≤ 4 after 1 min	5(15)	
< 7 after 5 min	7(21.2)	

All live birth were analyzed, *n* = 32. SGA: small for gestational age, AGA: adequate for gestational age

### Maternal complications in gestation

Five women (20.8%) developed preeclampsia resulting in caesarean section at a minimum of 26^2/7^, 29^4/7^, 32^3/7^,34^4/7^ and 35^0/7^ gestational weeks, respectively (Table 3**).** Gestational diabetes was detected in 2 cases (6.2%, Table 3). Interestingly, none of the patients after living donor transplantation presented with gestational hypertension, preeclampsia or gestational diabetes (data not shown). There was no case of maternal death in the cohort during pregnancy or within the first two years postpartum**.** In six women declining in kidney function and in two worsening of proteinuria were causes for delivery.

### Graft outcome

**Fig. 2:**
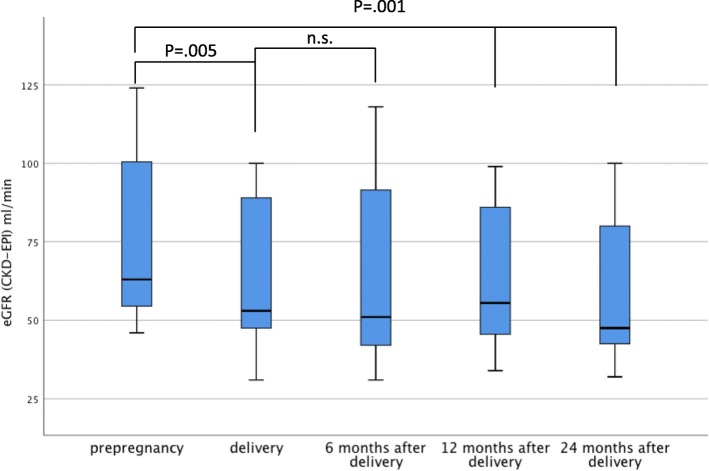
Kidney function during pregnancy and follow-up

Creatinine before pregnancy was 1.2 mg/dl in median (range 0.6–3.1) and GFR _**(CKD-EPI)**_ 57 ml/min/1.73m^2^ in median (IQR 52, 75 ml/min/1.73m^2^), respectively (Table 3). At delivery, GFR_**(CKD-EPI)**_ was significantly lower ((49 ml/min/1.73m^2^), IQR 37, 59 ml/min/1.73m^2^, *p* = .005) compared to pre-pregnancy values (Table 3**;** Fig. 2). Out of these patients, in 13 patients (46.4%) loss of GFR at delivery compared to pre-pregnancy values was more than 10 ml/min. Whereas, in 19 patients the loss of GFR was < 10 ml/min compared to pre-pregnancy values. At 6 months after delivery median GFR _**(CKD-EPI)**_ was 48 ml/min/1.73m^2^ (IQR 37, 60 ml/min/1.73m^2^). One year after delivery and at last follow-up median GFR _**(CKD-EPI)**_ remained lower compared to pre-pregnancy levels 50 ml/min/1.73m^2^ (IQR 39, 69 ml/min/1.73m^2^). GFR _**(CKD-EPI)**_ at 24 months after delivery was lower than the all other GFR _**(CKD-EPI)**_ values at delivery, and at 12 months after delivery there was no significant difference (Table 3). Proteinuria at delivery was in median 335 mg/g creatinine (Table 3).

Two graft losses were observed within one year after delivery, that occurred at intervals of one and 12 months, respectively (Table 3). Resulting in 92.8% 2 year graft survival after delivery. One patient had already poor kidney function before pregnancy with low GFR _**(CKD-EPI)**_ of approximately 17 ml/min. This patient experienced graft loss at one month after delivery corresponding to 9.3 years after transplantation. The kidney biopsy showed severe interstitial fibrosis, tubular atrophy and severe vascular damage. 14 months later she received a second transplant from her mother. The other patient experienced graft loss 12 months after delivery (6.1 years after transplantation), resulting in chronic graft nephropathy and thrombotic microangiopathy in the kidney biopsy.

Two patients after kidney-pancreas transplantation delivered three babies. They were delivered by caesarean section due to FGR at 30^4/7^, 32^2/7^ and 34^0/7^ weeks of gestational age, respectively. Both, pancreas and kidney graft function were stable during pregnancy and at the follow-ups 13, 90 and 139 months after delivery and at 85 and 163 months after kidney-pancreas transplantation. Overall, GFR _**(CKD-EPI)**_ at two years was still worse than at date of delivery with lowest values collected at 6 months.

### Fetal outcome and development

**Fig. 3:**
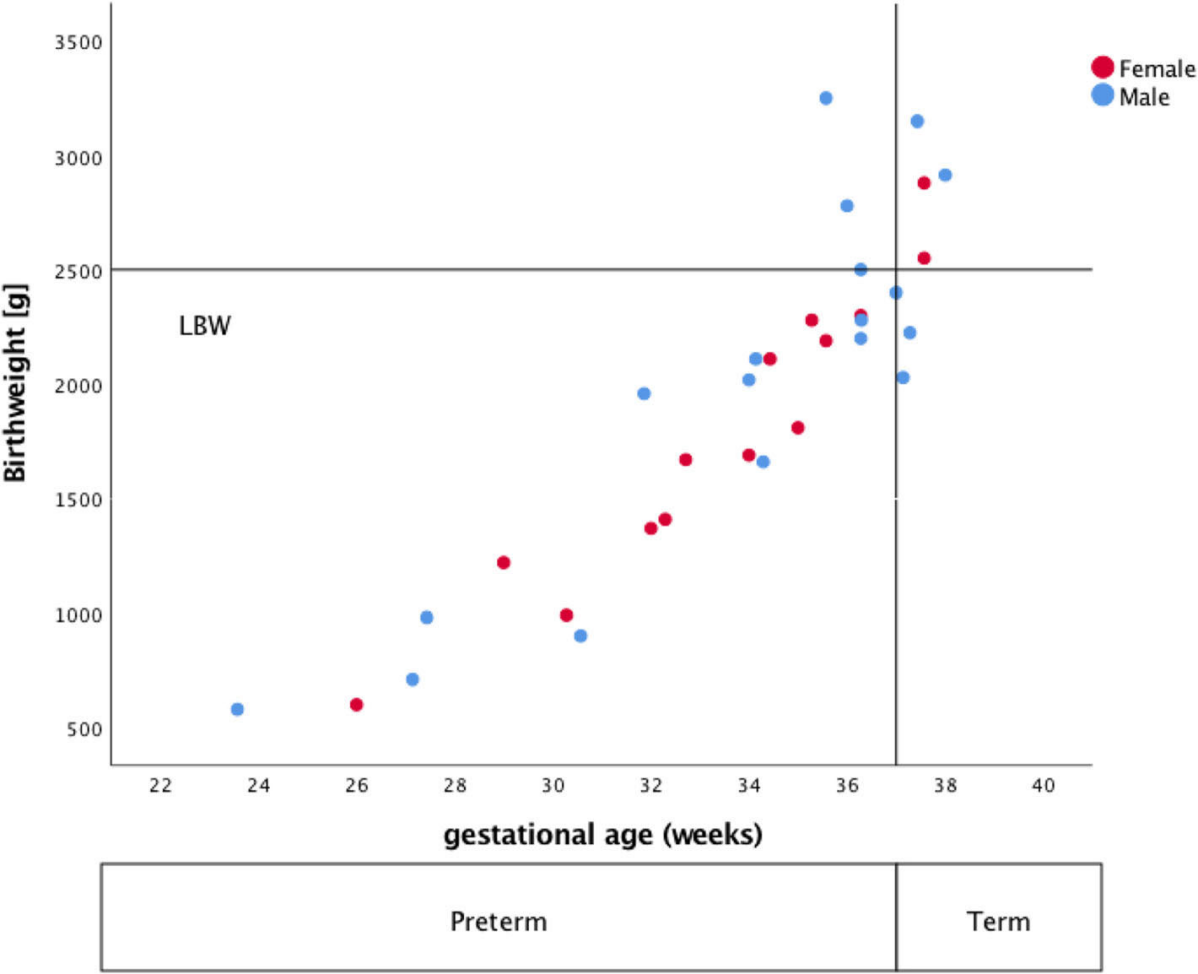
Birthweight and gestational age

The majority of the children (*n* = 25) were born prematurely (78.2%) and the gestational age was 34.7 weeks in median (IQR 31.9, 36.3 weeks; Table 4**,** Fig. 3). Reasons for preterm delivery were as follows: thereof 16 were caused by maternal indications, nine with contractions/ bleeding and seven with deterioration of graft function. Six patients had preterm delivery based on fetal indications: thereof four patients caused by pathological fetal heart rate and two patients due to growth arrest in combination with FGR. Three patients had preterm delivery based on combined fetal and maternal indications with pathological fetal heart rate pattern and contractions/ bleeding. Most often moderate-to-late premature babies (32^0/7^–36^6/7^ gestational weeks) was accounted for 53.1% of births; 21.8% were born > 37 gestational weeks. Characteristics of children are shown in Table 4. One baby died on the first day of his life due to extreme prematurity (23^5/7^ weeks). Birth weight was 2.030 g in median (IQR 1380 g, 2375 g; Table 3). The majority of children (75.7%) had low birth weights (< 2.500 g) (Table 4, Fig. 3).

The majority of children (71.8%) had a cord blood gas within a normal pH (> 7.2) range (Table 4). 25% had a pH between 7.1 and 7.2 (Table 4). Only 7/32 (21.1%) children had a low Apgar score < 7 five minutes after birth. Weight, length and head circumference at birth is shown subdivided in term and preterm delivery in Table 4.

### Childrens development from birth through age of 2 years

Response rate´ was 92.8%.

Evaluation of anthropometric measures 12 and 24 months after birth, subdivided in preterm and term at delivery, is shown in Table 5. Analysis of weight, length and head circumference was performed on children with an existing follow up of 24 months. Finally, 65.6% of children had complete dataset at 24 months (Table 5**,** Table 6). At birth significant differences were detected between the term and preterm group for weight/ length and head circumference (Table 5). 12 months after birth the differences in groups for weight/ length and head circumference had decreased and made no further significant difference (Table 5).

**Table 5:** Anthropometric measures at birth, at 12 and at 24 months of age in preterm and term born babies

Parameter	Preterm	Term	P value
Birth (median, IQR),			
Weight, g	1.810(1105, 2240)	2.550(2225, 2915)	0.002
Length,cm	43.0(37.0, 46.0)	48.0(46.0, 50.0)	0.006
Head circumference, cm	31.0(27, 32.5)	33(32.0, 34.0**)**	0.011
12 months (median, IQR),			
weight, g	8760(7670, 9570)	9180(9090, 9992)	0.704
length, cm	74.0(72.0, 75.5)	74.1(73.3, 76.3)	0.71
Head circumference, cm	46.0(45.0, 46.7)	46.0(46.0, 49.0)	0.365
24 months, (median, IQR)			
weight, g	11.800(10.950, 13.250)	11.900 (11.600, 12.900)	0.769
length, cm	84.0(83.0, 87.7)	86.0(84.5, 88.3)	0.308
Head circumference, cm	48.2 (47.5, 49.5)	49.5(48.5, 49.7)	0.410

Preterm less than 37 gestational weeks, Term ≥37 gestational weeks until 42 gestational weeks. Values are presented as median and interquartile range

**Table 6:** Weight-for-age, length-for-age and head circumference-for-age z-scores at birth, 12 and 24 months

Variables	Z-score
Weight (IQR)	
Birth	−1.04(−1.43, −0.54)
12 months	−0.375(−0.82, 0.1)
24 months	−0.31(−0.94, 0.32)
Length (IQR)	
Birth	−.79(− 1.43, − 0.15)
12 months	− 0.07(− 0.69, 0.28)
24 months	−0.78(− 1.15, − 0.17)
Head circumference (IQR)	
Birth	−.65 (− 1.4, 0.0075)
12 months	− 0.28(− 0.74, 0.51)
24 months	−0.05(− 1.1, 0.36)

Values are presented as median and interquartile range

**Fig. 4:**
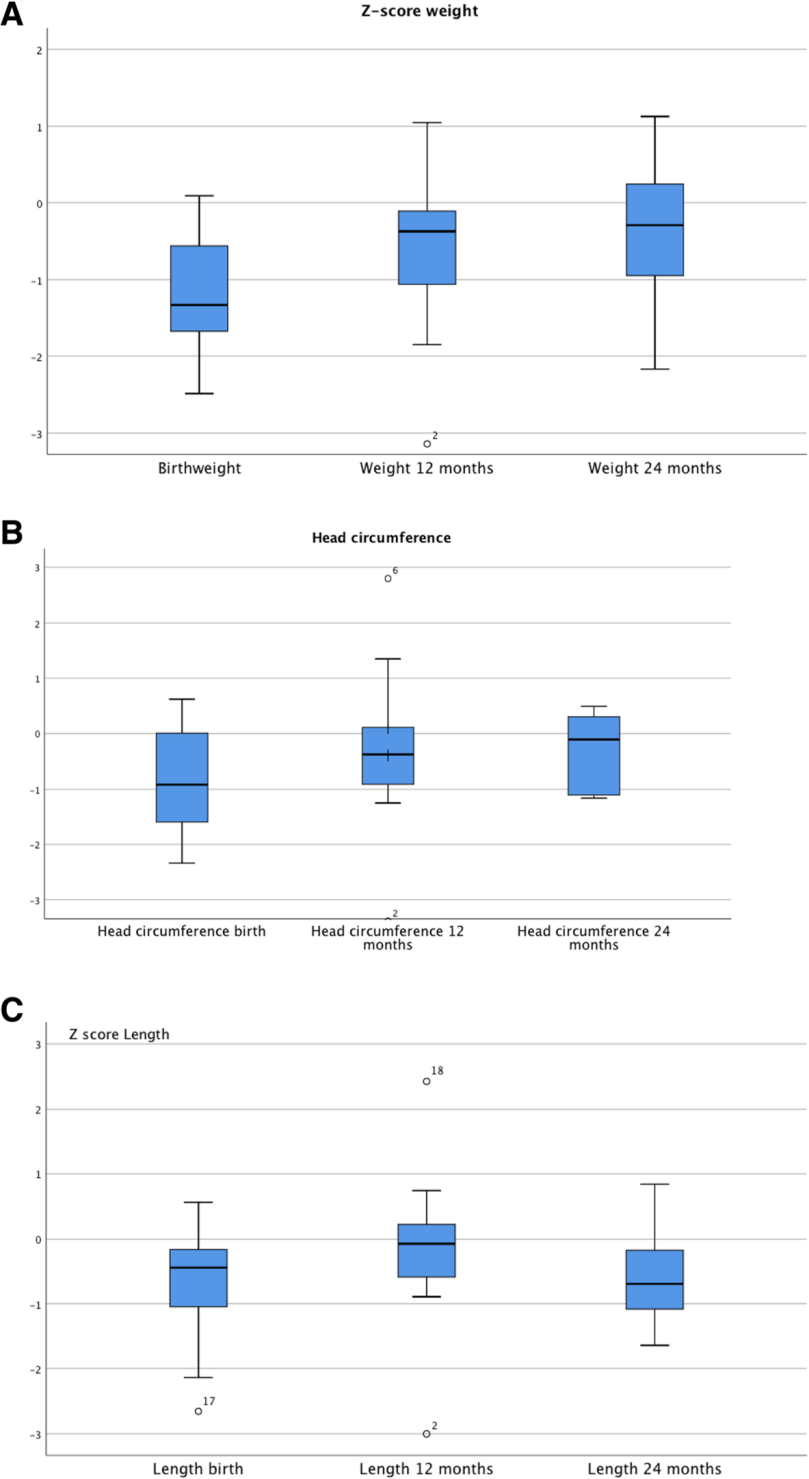
**a**-**c** Z score weight (**a**), head circumference (**b**), length (**c**)

**Fig. 5:**
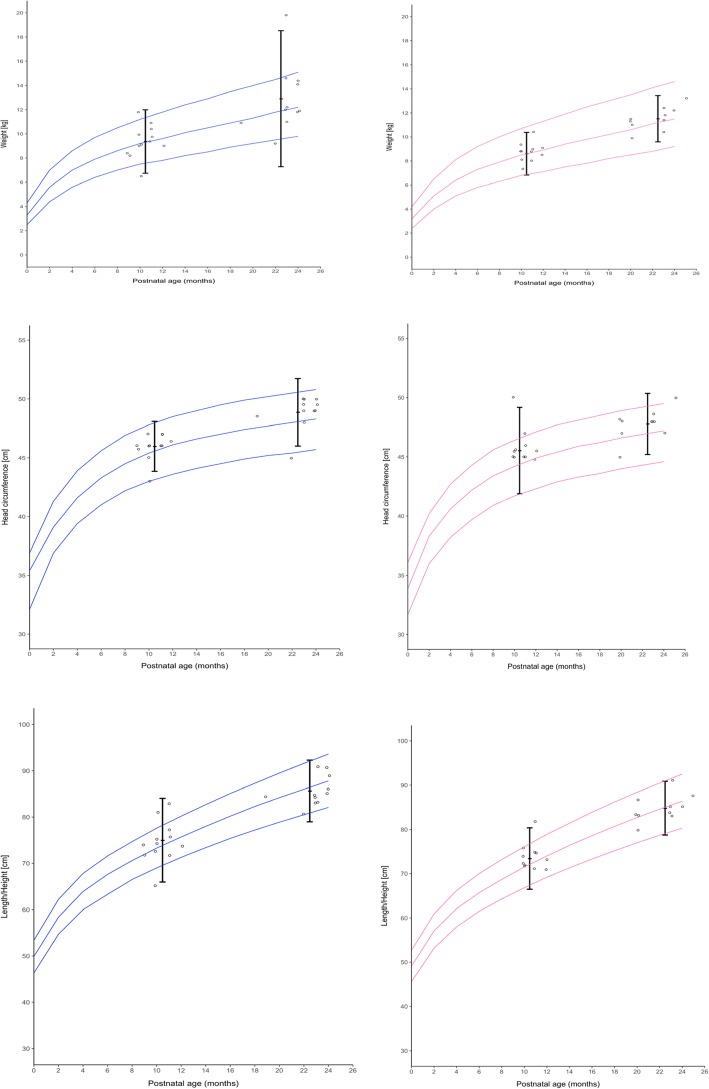
Anthropometric data. Development of children at 12 and 24 months of age. Values are overlapped onto the 3rd, 50th and 97th percentiles of the World Health Organization Child Growth Standards, girls lines in pink and boys blue lines. For preterm born children adjusted for calculated birth date was used

Differences tended to decrease after 12 and even 24 months of age in mean weight-for age, length-for-age and head circumference-for-age z-score (Table 6, Fig. 4a-c); at that time results have no significant difference between both groups regarding anthropometric data (Fig. 5).

The following abnormalities were observed at birth: microcephaly, microsomia and cryptorchism in one child and one child presented with strabism and bronchial asthma. The physical exam was normal in 93.75% (30/32) at 12 months and in 95.2% (20/21) at 24 months. In two very prematurely born children, mild neurological deviations were noted at one year of age, muscle tone disturbances in lower limbs and slightly reduced muscle tone in lower limbs, respectively. The respective findings had decreased in severity at 24 months of age. Noteworthy, their deficiencies were clearly found to have improved after two years of age. This was documented in the children’s medical records and was confirmed by neurological examination.

## Discussion

It is well recognized that risk of maternal and neonatal pregnancy-related complications in women having undergone kidney transplant is significantly increased when compared to general population [7]. Our study in 28 recipients confirms the high rates of obstetric complications in women following transplantation. To elucidate the developmental perspective of their children rather than to merely list and describe maternal factors, we further investigated the mid-term outcomes that were assessed by the pediatrician and were supplemented by a questionnaire sent out to parents. Taken together, outcome and development of children was found to be encouraging even though initial incidences of prematurity and low birthweight initially were substantial.

In our series, prematurity rate of 78% was higher compared to previous reports with incidences between 45 and 56% [2–5, 7] and especially when compared to a preterm delivery rate in the general population in Germany in 2016 with 8.6%. However, only one out of 32 infants analyzed died due to complications from extreme prematurity. Of note, predominantly moderate-to-late premature (32^0/7^–36^6/7^ gestational weeks) births were observed due to acute pregnancy-related causes such as acute placental insufficiency, severe preeclampsia, intrauterine growth restriction with abnormal fetal heart rate tracing, infections like chorioamnionitis or deteriorating kidney graft function.

Generally, preterm children, even if being late preterm (34^0/7^–36^6/7^gestational weeks) are at a higher risk for mortality and impaired neurodevelopmental outcomes such as cerebral palsy, mental retardation as well as more behavioral abnormalities than term-born children [21]. Therefore, it is of crucial importance to avoid even non-spontaneous late preterm birth to reduce not only immediate neonatal but also complications at later age. This necessitates consistent therapy of existing and defined risk factors. As recently reported, renal allograft recipients have a 13-fold higher risk of preterm deliveries and also a 5-fold high risk for small-for-gestation babies compared to the general population [22].

Birth weight was significantly lower in preterm compared to term-born babies. Nevertheless, despite a high rate of small-for-gestational age (31.5%) infants in our series, at 12 and 24 months all children had both adequate weight and height for age. This confirms results from a first large prospective controlled study in children born to transplant recipients [23] but is in stark contrast to another study examining children only at a median of eight years [24]. This suggests that retardation might still occur years after a seemingly normal development.

In our study, we acquired data until the age of two years, when the weight and height as well as head circumference differences compared to general population had decreased over time. Only one child was still small for age (Fig. 4a-c). We observed only in two very prematurely born children, mild neurological deviations at one year of age. The respective findings had decreased in severity at 2 years of age which is consistent with another report [23]. Interestingly, Nulman and coworkers found no differences in neurocognitive nor behavioral outcomes between eight year-old children exposed to cyclosporine in utero versus age-matched controls from healthy mothers [24]. In light of these findings it is difficult to make recommendations for general population about neurological outcomes at the age of 12 months due to the small sample size and the retrospective design.

In accordance to the report of Sibanda et al. [3], the rate of caesarean deliveries in this study was as high as 73.5% due to worsening of hypertensive disorders/ preeclampsia and deteriorating in kidney function while in the background population, 30.5% of all pregnant women in Germany deliver by caesarean section [18]. However, prognosis for mothers and children is nevertheless favourable.

Optimal timing might affect the outcomes of pregnancy, the maternal graft function and maternal comorbidities. We could show that premature birth rate, low birth weight and caesarean section rate were lower in the current study in pregnancies between two and five years after transplantation. A recent retrospective study of 729 pregnancies revealed an increased risk of graft loss during the first and second year after transplantation whereas pregnancy in the third year was no longer associated with an increased graft failure rate [25]. In accordance to our data, Mohammadi and coworkers showed that one-third of patients had deterioration in graft function during pregnancy and of those 63.2% did not return to baseline [8].

It still needs to be confirmed, whether our data suggesting increasing incidences of prematurity, low birthweight, caesarean section and preeclampsia in the group of women with an interval of > five years from transplant can be generalized. Interestingly, a recent report showed a high rate of preeclampsia but no association with long-term renal dysfunction [26].

In our analysis childrens´ development was followed up to the age of two years. However due to prolonged in utero exposure to a large variety of immunosuppressants, adverse effects occurring much later cannot be fully excluded. Specifically, cardiovascular and renal disease might be of importance [27, 28]. To gain more insight, children born to mothers receiving immunosuppression for other reasons than transplant could be followed to increase cohort size [29].

Taken together, in this complicated situation with multiple underlying factors affecting maternal and fetal outcome these data indicate an encouraging outcome provided that strict controls in close intervals be performed. The impact of sustained deterioration of kidney function following pregnancy requires further research.

## Conclusion

Pregnancies following kidney or kidney-pancreas transplantation are associated with a significant rate of prematurity and low birth weight babies and should therefore be considered as “high risk”. Development of the children up to 2 years after birth was found to be good and age-appropriate in our study. Affected women should be managed in tertiary care obstetrics centers working in a tight multidisciplinary cooperation with transplant physicians, i.e. nephrologists, diabetologists, and neonatologists. We are currently setting up a nationwide registry for pregnancy following solid organ transplantation to prospectively evaluate future cases. This may help to improve performance and outcome of both mother and their children.

## Data Availability

The data generated and used in the analysis of this study are included in this published article. Additional data is available from the authors upon reasonable request.

## Abbreviations

ELBW: extremely low birth weight
ESRD: End-stage renal disease
FGR: fetal growth restriction
GFR: glomerular filtration rate
LBW: low birth weight
MDRD: Modification of diet in renal disease
MPA: mycophenolic acid
SGA: small for gestational age
VLBW: very low birth weight
WGA: weeks of gestational age
